# Barriers to healthcare access and continuity of care among Ukrainian war refugees in Europe: findings from the RefuHealthAccess study

**DOI:** 10.3389/fpubh.2025.1516161

**Published:** 2025-04-02

**Authors:** Przemyslaw Kardas, Iryna Mogilevkina, Nilay Aksoy, Tamas Ágh, Kristina Garuoliene, Marta Lomnytska, Natalja Istomina, Rita Urbanaviče, Björn Wettermark, Nataliia Khanyk

**Affiliations:** ^1^Medication Adherence Research Centre, Department of Family Medicine, Medical University of Lodz, Lodz, Poland; ^2^Institute of Womens' and Childrens' Health, Uppsala University, Uppsala, Sweden; ^3^Institute of Postgraduate Education, Bogomolets National Medical University, Kyiv, Ukraine; ^4^Department of Clinical Pharmacy, Faculty of Pharmacy, Altinbas University, Istanbul, Türkiye; ^5^Medication Adherence Research Group, Center for Health Technology Assessment and Pharmacoeconomic Research, University of Pecs, Pecs, Hungary; ^6^Syreon Research Institute, Budapest, Hungary; ^7^Pharmacy Center, Institute of Biomedical Science, Faculty of Medicine, Vilnius University, Vilnius, Lithuania; ^8^Institute of Health Sciences, Faculty of Medicine, Vilnius University, Vilnius, Lithuania; ^9^Department of Pharmacy, Faculty of Pharmacy, Uppsala University, Uppsala, Sweden; ^10^Faculty of Medicine, Vilnius University, Vilnius, Lithuania; ^11^Department of Pharmacy, Danylo Halytsky Lviv National Medical University, Lviv, Ukraine

**Keywords:** Ukraine, war refugees, healthcare access, Temporary Protection Directive, chronic conditions, barriers to healthcare, refugee healthcare needs, European healthcare systems

## Abstract

**Introduction:**

The Russian invasion of Ukraine displaced over 14 million people. By 2024, around 6 million Ukrainian refugees settled in Europe under the EU Temporary Protection Directive, providing permit of residence, work and health care. This influx strained European healthcare systems, particularly in addressing acute injuries. As the stay of refugees in EU countries prolongs, the management of chronic conditions becomes increasingly important. However, there is limited information available about Ukrainian refugees' access to various healthcare services.

**Aim:**

The aim of this study was to evaluate perceived accessibility of healthcare services in Europe for Ukrainian war refugees and to identify barriers to healthcare access, in order to inform improvements in healthcare provision.

**Methods:**

A cross-sectional online survey was conducted across Europe from July 2023 to April 2024, targeting adult Ukrainian war refugees. Survey explored areas defined as key health care needs. Descriptive, parametric and non-parametric statistical analysis methods were employed in data analysis.

**Results:**

Of 659 respondents, 550 (83.4%) were included in the final analysis due to having reported need to use healthcare services in the past year. The most prevalent needs included dental care (82.9%), prescription medication (81.6%), care for acute (78.4%), and chronic conditions (64.0%). Perceived access to care varied across services, with vaccinations rated highest, while chronic condition care rated lowest. Around ¼ of respondents reported that they had to temporarily return to Ukraine for services not available in the countries where they stayed, these being mostly dental and gynaecologic care. The most prevalent barriers reported were long waiting times (64.2%), information barriers (55.5%), and high service costs (49.1%).

**Discussion:**

The survey identified several barriers in the access to healthcare system for Ukrainians, particularly for chronic conditions care. Some barriers may be subjective, relating to limited access to information. However, others point to potential shortcomings within national healthcare systems, suggesting areas that require further review and improvement.

**Conclusions:**

Addressing language barriers, improving information dissemination, and enhancing chronic condition management were identified as crucial for improving healthcare access for Ukrainian war refugees. Coordinated strategies are needed to support refugees and ensure the sustainability of host healthcare systems.

## Introduction

Due to the ongoing Russian invasion of Ukraine that began on February 24, 2022, over 14 million Ukrainian citizens (nearly 35% of the population) were forced to flee from their homes, seeking refuge both within Ukraine and beyond (1). Many of them migrated to other countries, mostly the European Union member states. As of early 2024, ~6 million of Ukrainian war refugees were dispersed in various European countries (1).

Such a huge influx of people over a short time posed unprecedented challenges to the European healthcare systems, particularly in neighboring countries such as Poland, Germany, the Czech Republic, and Lithuania (2). Apart from the obvious necessities of providing refugees with acute care due to war and transportation-related conditions, European healthcare systems have always needed, and still need to respond to the huge demand for other services related to non-acute conditions (3). Of importance is that among individuals who reported needing healthcare in 2022, the year the war hostilities began, chronic conditions were the second most commonly cited reason, accounting for 29–40% in studies by the United Nations High Commissioner for Refugees (UNHCR) and the World Health Organization (WHO) (4, 5). This is not surprising as chronic non-communicable diseases (NCD) are highly prevalent among adult Ukrainians. Around one-third suffer from hypertension and 7% from diabetes (6). Therefore, it is obvious that the longer they stay in the host countries, the more important it becomes to provide care required in their chronic conditions.

Effective disease management requires constant access to medicines and timely healthcare, i.e., resources that are often scarce for many war refugees. Upon arrival in host countries, most of them lack medical documentation, valid prescriptions and adequate supplies of essential medications (6). Those suffering from chronic kidney failure, for example, need regular dialysis, while refugees with cancer face even greater challenges. Interruptions in their treatment can exacerbate the spread of cancer, yet the care they should receive is often complex, available in specialized centers only, and requires in-depth knowledge of the disease process (7). Older adult refugees and individuals with disabilities, who often suffer from multiple health conditions, are particularly vulnerable. Unfortunately, they are frequently given low priority in healthcare settings and encounter numerous barriers that restrict access to appropriate care (6).

The immediate response of the European community enabled millions of Ukrainian war refugees to access healthcare in the countries of their temporary stay. For the first time, the European Commission (EC) activated the Temporary Protection Directive 2001/55/EC, allowing Ukrainian citizens fleeing their country to receive immediate healthcare (6). In the European Union (EU) member states, several initiatives were quickly mobilized. Municipalities and local health systems received tailored guidance on addressing health needs of Ukrainian refugees, and various platforms were created to inform them of their rights under this Directive (8). Two days after the Russian invasion, Poland offered all refugees escaping the war access to the same healthcare as that provided to Polish citizens under their National Health Fund (6, 9). In some other countries, such as Lithuania and Sweden, access was more limited and not as immediate (10, 11).

However, lessons learned from the COVID-19 pandemic, which also recently struck Europe, revealed that many European countries were not fully prepared to maintain chronic condition care in such challenging circumstances (12). Therefore, it is crucial to evaluate the actual performance of the declared support. This involves early identification of specific medical services required by war refugees, especially in long-term therapies, and adapting healthcare services accordingly. Additionally, gathering and analyzing feedback from the refugees themselves is essential to accurately assess how well the provided support meets their needs.

Currently, limited information is available about Ukrainian refugees' access to healthcare services in Europe. Therefore, the aim of this study was to evaluate perceived accessibility of the healthcare services for Ukrainians under the Temporary Protection Directive and identify barriers to healthcare access and continuity of care, and thus inform improvements in healthcare provision to Ukrainians and during crisis in general.

The need to address the challenges faced by Ukrainian refugees in European countries brought the idea of a comprehensive survey. It was then turned into a study entitled “Ukrainian War Refugee Access to Non-acute Healthcare Services Across Europe (RefuHealthAccess Europe)”, which was developed under the framework of the ENABLE COST Action. ENABLE (European Network to Advance Best Practices & Technology on Medication Adherence, CA19132) is a Europe-wide scientific collaboration which aims to transform healthcare systems toward better adherence support, thus helping patients regularly receive their evidence-based therapies and achieve better health outcomes. Currently, ENABLE gathers more than 200 members from 40 countries, including 39 European ones, as well as Ukraine and Israel (13).

## Methods

### Survey design

This cross-sectional study comprised two main phases: (i) designing of the tool, i.e., the study questionnaire, and (ii) collecting data on the European level with online survey conducted among Ukrainian war refugees.

This survey questionnaire was developed in a stepwise process to assess accessibility to key healthcare services among Ukrainian refugees across European countries. Following the initial discussions at the ENABLE meeting in Zagreb (March 30–31, 2023), the overall focus of the survey was defined, and a provisional shortlist of potential dimensions was created based on similar studies conducted in this area (4, 5). This list served the creation of a technical survey in which research team expressed their preferences regarding the inclusion of a particular dimension in the final version of the questionnaire, and could suggest additional ones. As many as 24 various health services were assessed according to the 5-point Likert scales, with 1 point awarded for the lowest priority, to 5 points for the highest priority. Twelve individual items reached the average value of 4.0 points or above and they were provisionally accepted. The top-ranked items were further reviewed and clustered to reduce the length of the survey and minimize the burden on participants, which resulted in the final approval of 11 key healthcare services: care for acute conditions, chronic diseases, mental health, dental care, child and older adult care, cancer care, sexual and reproductive health, vaccination, cancer screening, and prescription medicines. Subsequently, based on these items, the first draft of the questionnaire was developed. The phrasing of questions and potential answers were discussed and agreed upon by the study partners of whom all had medical education. A thorough yet fast discussion resulted in the final survey. The original survey questionnaire was developed in English and then it was translated into Ukrainian by two native language researchers of whom one prepared the translation and the other checked it carefully and back-translated the text. Before the survey was finally opened to the respondents, a pilot study in a limited number of Ukrainian native language speakers had also been conducted. They were asked to go through the whole questionnaire and assess its readability, ease of navigation of its online version, and overall performance. Feedback collected from those volunteers was analyzed, and relevant modifications were introduced, if deemed necessary. This procedure ensured that the final version of the survey was easy to use, which was aimed at increasing the response rate. The simple wording and accessible language translation allowed participants from different backgrounds to complete the questionnaire using their phones or other handheld devices, laptops, desktops, etc.

The resultant final version of the questionnaire (Supplementary material 1) used in this survey contained 26 questions with relevant skip options, to allow for a fast completion by participants. It covered the following issues:

1. Socio-demographic characteristics of the participants: gender, age, education, country of residence, country of origin, residence status (e.g., residence permit, temporary documents, undocumented), housing situation (reception center, house, street etc.), number of people living under the same roof, living with children.
2. Need to access various types of healthcare services (among the survey participants or any member of their families) in host countries in the previous 12 months.
3. Accessibility of medical services the respondents needed to use in the previous 12 months in host countries (as reported by the survey participants and their family members)
4. Perceived barriers toward medical services.

At the end of the questionnaire, the participants were requested to use the “snowball method” to further disseminate the survey. Additionally, a useful link was given to find information on the services provided by the European Union across national healthcare systems in Europe, including some resources available in Ukrainian.^1^

### Data collection

A cross-sectional anonymous online survey targeting Ukrainian war refugees was conducted with the use of specialized service (SurveyMonkey.com) across Europe from July 12, 2023 to April 16, 2024, by multi-channel invitation and snowball sampling.

The survey invitation was shared through the ENABLE network using internal communication channels like e-mail to promote participation and encourage further distribution among Ukrainian refugees in various countries. ENABLE members targeted local stakeholders, including non-governmental organizations (NGOs), medical centers, and refugee groups. Additionally, e-mails and the ENABLE COST Action Facebook page were used to reach a broader public, organizations, policymakers, and refugee groups. Various organizations, such as WHO, UNHCR and the European Commission, were also contacted to assist in survey dissemination. At the end of the survey, the participants were encouraged to spread the questionnaire further among Ukrainian refugees. A snowball sampling helped to reach refugees who otherwise might not have had a chance to access the survey. The goal was to invite these vulnerable groups through various networks and encourage participation to capture their perspectives.

Participation was voluntary. Online surveying system settings were set to block multiple entries from the same device. No incentives for participation were offered. At the beginning of the survey, before giving informed consent, the participants were advised of the objectives, data usage and storage, and expected use of the results.

### Survey participants and inclusion criteria

The target survey participants were war refugees from Ukraine, aged 18 years and older, residing in any member state under the temporary protection, as set by the relevant EU Directive. However, the research design did not include specific criteria to determine refugee status as a requirement for participation in the survey. The participants of the survey were free to determine whether they considered themselves to be a “war refugee”, and no formal proof of their status was requested. To ensure unbiased feedback, the final analysis was limited to the respondents who indicated that they themselves or their family members had needed at least one of the targeted healthcare services.

### Data management and statistical analysis

Full data anonymity was provided. No IP addresses of computers/mobile devices used to fill in the survey were processed. Although in principle the survey collected sensitive information on age, sex, country of origin and country of residence, and legal status, it was highly unlikely that the participants could be identified using such information in combination. Like the whole participation in the survey, all information and answers to specific questions were provided voluntarily. Each participant had an option of omitting questions which they were not willing to answer.

The main construct of the study, i.e., the need to access different types of health services, was operationalized through a positive response to the relevant survey question. For example: “In the past 12 months, in the country of your current stay, did you or another person in your household need to access health services for any chronic illness?”

Free text survey responses were translated into English, categorized and clustered for better clarity. Perceived access score was calculated with values ascribed to Likert scale answers (where −2 corresponds to “very poor access”, −1 to “poor access”, 0 to “neither poor nor good access”, 1 to “good access”, and 2 to “very good access”). Perceived access to healthcare services was categorized by calculating mean access scores based on Likert scale responses. The average scores for each healthcare service were analyzed and grouped into four clusters to facilitate interpretation: “very good access” (≥0.75 mean score), “fairly good access” (0.25–0.74 mean score), “satisfactory access” (0–0.24 mean score), and “unsatisfactory access” (< 0 mean score). This clustering method allowed for a structured comparison of access levels across different healthcare services, enabling a clearer identification of disparities in service availability.

Descriptive statistical analyses were performed to summarize the data. Specific countries were included in the benchmarking analysis if they accounted for at least 10% of the total survey responses. The Kruskal-Wallis test and Chi-square test were applied where appropriate. The analyses were conducted using SPSS and R statistical software.

### Ethics

The study was approved by the Ethical Committee of the Medical University of Lodz, Poland, on June 13, 2023 (Approval No. RNN/177/23/KE) and by the Swedish Ethical Review Authority on September 7, 2023 (explanatory letter Dnr 2023-.03597-01). The survey was conducted in accordance with the ethical standards, with full anonymity and confidentiality.

## Results

As many as 659 responses to the survey were collected among which 594 responses were provided by individuals identifying themselves as adult Ukrainian war refugees. The final analysis included 550 individuals who reported that they themselves or their family members had needed or been provided with at least 1 of the 11 medical care services included in the previous 12 months, in their current country of residence.

Characteristics of the analyzed cohort was presented in Table 1. Ninety percent of the respondents were female, over three quarters of the group had higher education. The mean age (SD) was 40.1 (10.9) years. The respondents had been living in the country of their current stay for an average of 17.1 (5.3) months. A majority of the respondents (89.3%) lived in a house or apartment, and only a small minority reported living in an asylum center, a refugee camp or on the street (10.0 and 0.4%, respectively). A vast majority of the respondents reported to possess temporary protection documents (96.2%). They lived under the same roof with 3.9 (10.5) people, and only 14.9% of the respondents (82 persons) declared that they had been living alone. As many as 373 (67.8%) of the respondents indicated living together with children, with an average number of children being 1.6 (4.3) per respondent. A vast majority of the respondents lived in the three countries, namely Sweden, Lithuania and Poland (44.7%, 27.6%, and 16.5%, respectively).

**Table 1:** Characteristics of the survey respondents included in the final analysis.

**Variable**	***N* (%)**
**Age (years)**	
18–39	281 (51.1)
40–64	246 (44.7)
65+	20 (3.6)
Missing data	3 (0.5)
**Gender**	
Female	495 (90.0)
Male	51 (9.3)
Other/not willing to provide	3 (0.5)
Missing data	1 (0.2)
**Education**	
Higher education	432 (78.5)
Secondary special education	85 (15.5)
Secondary education (from 10 to 11 grade)	22 (4.0)
Incomplete secondary education (9 grade)	5 (0.9)
Other	6 (1.1)
**Length of stay in the current country (months)**	
1–12	93 (16.9)
13–18	203 (36.9)
19+	250 (45.5)
Missing data	4 (0.7)
**Place of living**	
House/apartment	491 (89.3)
Asylum center/refugee camp	55 (10.0)
On the street	2 (0.4)
Other place	2 (0.4)
Missing data	1 (0.2)
**Residence status**	
Possess documents for temporary protection	528 (96.2)
Possess documents for permanent residence	13 (2.4)
Does not have documents for a legal stay in this country	2 (0.4)
Other	6 (1.1)
**Number of people living under the same roof**	
Mean (SD)	3.9 (10.5)
Median	2.0
**Number of children (0-18 years) living with the respondent**	
Mean (SD)	1.6 (4.3)
Median	1.0
**Host country**	
Sweden	246 (44.7)
Lithuania	152 (27.6)
Poland	91 (16.5)
Greece	21 (3.8)
Estonia	10 (1.8)
Other^#^	29 (5.3)
Missing data	1 (0.2)
Total	550 (100.0)

^#^Countries with no more than 2 responses were collectively presented under the “Other” category.

### Need for various health services across EU

**Figure 1:**
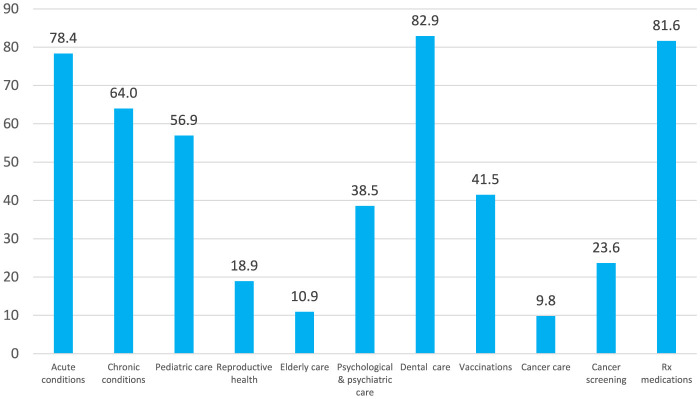
Percentage of survey participants reporting the need for various healthcare services within the last 12 months in their current country of residence, either for themselves or another household member.

The reported need for various health services is illustrated in Figure 1. When asked about a need to use healthcare services within the previous 12 months in their current country of residence, either in relation to themselves or a household member, 82.9% of the respondents indicated a need for dental care, followed by care for acute conditions (78.4%), chronic diseases (64.0%), and child care (56.9%). Around 40% of the respondents declared needs for vaccination (41.5%) and mental care (38.5%). The need for cancer screening was reported by one fourth of the respondents while sexual and reproductive health care by one fifth. The lowest number of the respondents reported needs for cancer and older adult care (9.8 and 10.9%%, respectively). As many as 81.6% of the study participants reported the need for prescription medications in the previous 12 months.

### Access to various health services across EU

**Figure 2:**
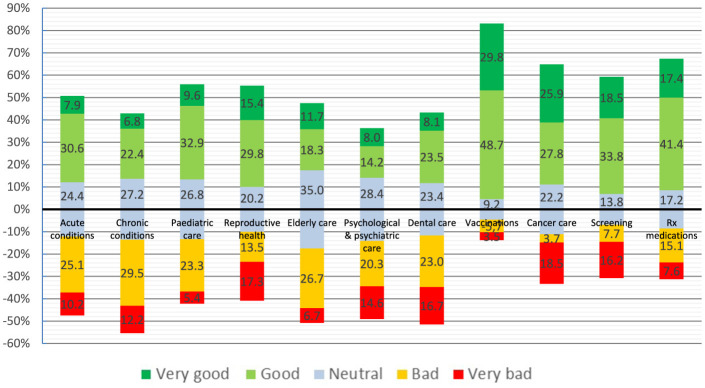
Perceived access to various health services (needed by survey participants or their household members) across the EU, as reported by the respondents. Numbers represent percentages.

Access to various health services, as reported by the study participants, varied significantly (Figure 2) and, based on the mean values of the calculated scores, could be grouped into four clusters as follows:

- Very Good: Access to vaccinations (0.95),
- Fairly Good: Access to prescription medications, cancer care and cancer screening (0.46, 0.39, and 0.30, respectively),
- Satisfactory: Access to pediatric care, reproductive health, older adult care and care for acute conditions (0.18, 0.12, 0.02, and 0.01, respectively),
- Unsatisfactory: Access to psychological and psychiatric care, care for chronic conditions and dental care (−0.19, −0.18, and −0.17, respectively).

Notably, perceived access to care for chronic conditions was reported as the poorest among all of services surveyed, with the highest percentage of the respondents (41.8%) rating their access as either “bad” or “very bad”.

As many as 130 respondents (23.6% of the total) declared that they had to return to Ukraine temporarily to access healthcare services that, to their understanding, they could not obtain in their current country of stay in the previous 12 months. Among these services, the most frequently mentioned were dental, gynecological and ophthalmological care reported by 66.2%, 26.2%, and 9.2% of the refugees, respectively (Table 2).

**Table 2:** Healthcare services that the respondents reported as unavailable in their current country of residence, leading to temporary returns to Ukraine within the past 12 months.

**Type of healthcare service**	***N***	%^*^
Dental care	86	66.2
Gynecology	34	26.2
Ophthalmology	12	9.2
Endocrinology	9	6.9
Orthopedics	7	5.4
Otolaryngology	6	4.6
Dermatology	4	3.1
Gastroenterology	4	3.1
Neurology	4	3.1
Other^#^	51	39.2

^*^Percentages are calculated based on a total of N = 130 respondents who provided a positive answer to this question. Note that many respondents indicated multiple services as unavailable.

^#^It also included various diagnostic procedures.

### Barriers to various health services across EU

When asked about the ease of obtaining necessary information regarding the structure and operational procedures of the local healthcare system in their current country of stay (e.g., from websites, telephone helplines, etc.), as many as 42.7% of the respondents found it either “very hard” or “hard”. Additionally, 51.8% of the study participants reported that information on the local healthcare system's structure and operational procedures was either not available at all or hardly available in the Ukrainian language.

**Figure 3:**
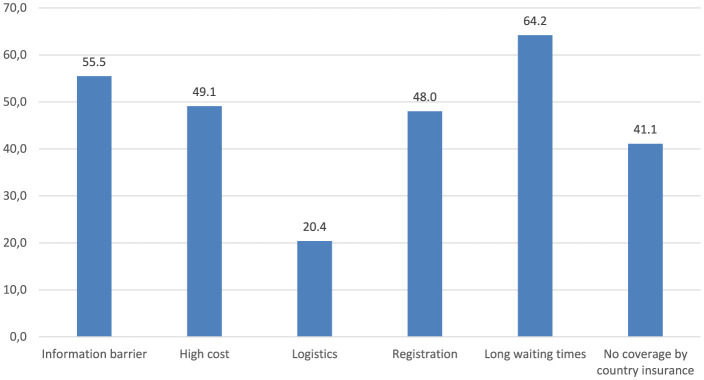
Barriers to various health services (needed by the survey participants or their household members) across the EU, as reported by the respondents. Figure presents the total percentage of the respondents who assessed a particular barrier as either “very important” or “important”.

Also, the respondents were asked to identify potential obstacles restricting access to healthcare services for themselves or their household members over the previous 12 months, with six different predefined obstacles provided for assessment. The most prevalent issues identified as “very important” or “important” were long waiting times (64.2%), information barriers (e.g., lack of information, language, cultural barriers) (55.5%), and high service costs (49.1%). These were followed by registration procedures (48.0%) and lack of coverage under the host country's national insurance (41.1%). Logistic difficulties, such as transport and distance, were the least frequently mentioned barriers, indicated by 20.4% of the respondents only (Figure 3).

### Access to various health services across benchmarked countries

Differences in access parameters across the benchmarked countries are illustrated in Supplementary material 2. A statistically significant difference across the countries was observed for access to cancer screening (*P* = 0.004), and a marginally significant difference for psychological and psychiatric care (*P* = 0.0536).

Similarly, although the percentage rates of the respondents declaring that they had to return to Ukraine temporarily for healthcare services in the previous 12 months varied slightly between Lithuania (17.8%), Poland (24.2%), and Sweden (28.0%), the differences were not statistically significant (Chi-square = 5.4291, *P* > 0.05). Details of the services the respondents had sought in Ukraine are presented in Supplementary material 3.

### Barriers to various health services in benchmarked countries

**Figure 4:**
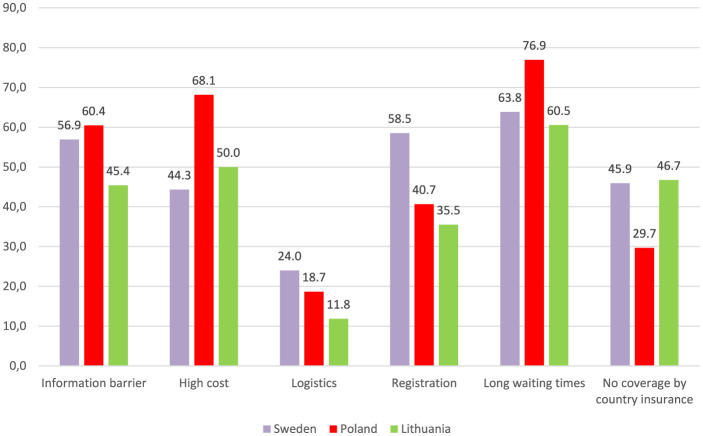
Distribution of the barriers to various health services in the benchmarked countries. Figure presents the total percentage of the respondents who assessed a particular barrier as either “very important” or “important”.

Figure 4 illustrates the distribution of barriers to various health services across the three benchmarked countries with the highest number of study participants, i.e., Poland, Sweden and Lithuania. The most prevalent barrier reported by the respondents in all the three countries was long waiting times, identified as either a “very important” or an “important” barrier by 76.9% of the respondents in Poland, 63.8% in Sweden, and 60.5% in Lithuania. The third most common barrier in these countries was information barrier, indicated by 60.4% of the respondents in Poland, 56.9% in Sweden, and 45.4% in Lithuania. However, the second most significant barrier varied among the countries. In Poland and Lithuania, it was the high cost of services indicated by 68.1 and 50.0% of the respondents, respectively, while in Sweden, registration problems were the second most significant barrier mentioned by 58.5% of the respondents.

## Discussion

This was a cross-sectional survey documenting the perceived availability of various healthcare services to Ukrainian war refugee across different EU member states. It assessed their access to care and identified perceived barriers, gaps, and informational shortcomings in the provision of necessary services. In particular, the survey aimed to uncover the challenges Ukrainian refugees in EU countries faced when trying to get access to long-term therapies for chronic conditions.

In the initial stages of their stay, war refugees primarily focus on addressing acute health conditions related to war hostilities and the migration process, such as traumas and infections. However, as their stay extends, management of NCDs becomes increasingly important. The shift in priorities is evident in the results of this survey that examines healthcare needs of Ukrainian refugees over the past 12 months spent in their current countries of residence. The survey highlights that dental care and treatment for acute conditions were among the most frequently reported needs, representing the “acute dimension” of healthcare. At the same time, a significant proportion of the respondents, despite their relatively young age, reported the need for prescription drugs (81.6%) and care for chronic conditions (64.0%), indicating the ongoing treatment and self-management of chronic diseases. These findings emphasize the critical importance of addressing the long-term healthcare needs of refugees.

Ensuring continuous access to medications and healthcare services for war refugees is essential. The WHO 2022 report calls for urgent global action to support the health of refugees and migrants, pointing at, among other things, the need to take care of their chronic conditions (14). In the case of Ukrainian war refugees, this particularly refers to cardiovascular disease (CVD), considering its high prevalence in Ukraine before the war (8, 15, 16). An analysis found that of the estimated 6.12 million Ukrainians who fled the country between February 24 and May 13, 2022, ~1,072,532 had CVD, 253,275 had diabetes and 40,011 had cancer (17). The available data particularly emphasize the need for enhanced healthcare systems to manage the high burden of chronic conditions in older adult refugees who are often affected by multimorbidity and require ongoing care (18, 19). Finally, NCDs are a critical concern even among child refugees. They not only require care for acute illnesses, screening procedures, and vaccinations but also consistent management of chronic conditions, such as insulin-dependent diabetes (20).

Considering the frequent need for care related to chronic conditions, the fact that access to these services ranked the worst among the 11 types studied is deeply concerning. Over 40% of the respondents assessed this access as either “bad” or “very bad”. It is worth mentioning that disrupted or inadequate access to screening and treatment significantly increases the risk of migrants presenting with advanced diseases and complications from non-communicable conditions, including cancers, which ultimately lead to higher morbidity and mortality rates (21, 22).

Interestingly, a large UNHCR study conducted in Poland as early as in 2022 found that 81% of individuals who reported healthcare needs were able to access the necessary care (4). Similarly, a WHO survey revealed that a vast majority (92%) of refugees in Poland who required medical care in 2022 were able to receive it (5). These findings seem to be in contrast with the results of our study. However, it is of note that unlike WHO and UNHCR surveys, which were performed in person, this study was an anonymous online survey. This could minimize the social desirability bias and allow the respondents to unveil their true problems with access to certain services. On the other hand, the convenience sampling method could introduce another bias since dissatisfied respondents are generally more motivated to share their opinions in surveys than those who are satisfied. Finally, when interpreting the observed differences, it is crucial to consider the growing importance of access to care for chronic conditions, which has become increasingly significant as the stay of refugees prolongs.

A particularly interesting insight emerges from the analysis of services that the respondents indicated as those unobtainable in their current country of stay, which prompted them to temporarily return to Ukraine. The number of such cases was strikingly high, reaching nearly 25% of the respondents. Being reported by two thirds of these people, dental care was the most frequently mentioned reason why refugees searched for care in their country of origin, primarily due to the unavailability of free services and the high cost of private care in other countries where they had stayed. Noticeably, according to recent OECD reports, only 13% of dental care spending is publicly funded in Lithuania, and 43% in Sweden (23, 24). Hence, in response to the limited accessibility of dental services, several dental companies in Lithuania have begun offering free services for Ukrainians (25).

On the other hand, out of the health services analyzed, access to vaccinations and prescription drugs was rated particularly well. This optimistic finding, however, may be at least partly explained by the fact that in both these dimensions, EU healthcare systems offer their citizens much more than the Ukrainian one. For instance, the number of compulsory free of charge vaccinations is much higher in EU countries, and the reimbursement of drugs is quite common, whereas in Ukraine it covers selected chronic medications only (3).

Limited access to information about local healthcare systems, particularly in Ukrainian, played a central role in refugees' negative experiences related to healthcare in their host countries. Unfortunately, our data prove that over 40% of the respondents found it “very hard” or “hard” to obtain necessary information, and more than 50% reported that information in Ukrainian was unavailable or difficult to find.

Certainly, language barriers played a significant role in this issue, as they are a major obstacle for migrants seeking access to health services (26). Non-native speakers struggle with service registration, communication with medical staff, and use of untranslated electronic tools. These barriers persist in medical settings where translation services are often unavailable or underused. Some migrants turn to private facilities for better language support, however, many cannot afford them. Still, language barriers seem to be only a part of the reason for the poor understanding of local healthcare systems, and the overall provision of this information to refugees could be optimized. This is especially true given that the survey respondents, who had been in their current country of residence for an average of 17 months, had ample time to use available information services and become familiar with structures and procedures within local healthcare systems.

Unfortunately, despite early warnings about the need for an adequate information system following the war outbreak (27), there still remains a significant barrier to access that results from a lack of awareness about available healthcare services, scope of health insurance, formal procedures (e.g., determining disability), and other critical knowledge (18). In consequence, Ukrainian war refugees often do not obtain essential information about their rights. In Lithuania, 40% were unaware of their entitlement to free medical services (28). In the UK, confusion over NHS services led to decline in mental health (29). Similar problems were observed in Poland, Slovakia and Germany, where language barriers and unfamiliarity with healthcare systems impeded access (30–32). In 2023, Lithuania's State Audit Office released a report on the challenges of refugee integration and healthcare access, emphasizing the need for improvement through greater local government involvement, stronger collaboration between the state, municipalities, and NGOs, and addressing gaps in communication and healthcare delivery (28). These findings highlight the need for targeted support to help refugees navigate healthcare and understand their entitlements.

Similar issues, particularly low understanding of local healthcare systems, may also explain why in our survey, many respondents identified high costs and lack of coverage by host country health insurance as major barriers. This needs to be commented upon in the context of the equal access that should theoretically be granted to Ukrainian war refugees in their host countries under the EU Directive, providing them with the same rights as local citizens. However, equal access does not guarantee free availability of every service. Out-of-pocket payments, particularly for pharmaceuticals and dental care, are high in Lithuania (23, 33), Poland (9, 34), and Sweden (24, 35). Moreover, in Sweden, Ukrainian refugees initially had limited access to certain services. As of November 2024, new regulations allow a larger group to register as residents, expanding their access to healthcare beyond emergency care (11).

An interesting result of our survey was that the respondents identified long waiting times as the most significant obstacle restricting access to healthcare services. This finding is in line with previous results of the UNHCR and WHO surveys (4, 5). However, long waiting times have been reported as one of the major issues in national healthcare systems across many countries, including the three countries benchmarked in this study. In Poland, for example, the average waiting time for specialist appointments increased to 4.3 months in 2024, up by 0.6 months from 2023 (36). Therefore, further research may be required to determine whether these long waiting times were indeed excessive, or if respondents had unrealistic expectations. Causative factors to be considered include a possible lack of understanding of a host country's healthcare procedures (e.g., whether a referral to a specialist is required) and their specific experiences with the Ukrainian healthcare system. Indeed, a study conducted among German general practitioners proved that one third of them faced difficulties in care provision due to refugees' expectations concerning offered services (37).

When interpreting the results of this survey, it is important to note that, although Ukrainian war refugees have been granted access to European healthcare systems through the activation of a relevant directive, EU member states vary significantly in the structure and operation of their healthcare systems. These systems may differ greatly from what Ukrainians are familiar with in their home country. Nevertheless, with only three countries included in the benchmarking analysis, the conclusions drawn from this exercise may be somewhat limited. These countries vary significantly from the perspective of Ukrainian refugees. Both Lithuania and Poland are neighboring countries with many linguistic and cultural similarities, which may presumably facilitate integration for refugees. In contrast, Sweden, a Nordic country, shares fewer commonalities with Ukraine. This cultural and geographic disparity may have contributed to the lower ratings of certain services in Sweden, such as management of mental health and chronic conditions, compared to the other countries. Nevertheless, the particularly poor assessment of dental care in Poland appears to be associated with different factors, most probably high costs, as many dental services are not available free of charge within the national healthcare system, and hence may be expensive in the private sector. Similar conclusions can be drawn from the benchmarking of country-specific barriers. The high prevalence of registration issues in Sweden is likely to reflect linguistic challenges, which are more pronounced in this country than in the other two analyzed.

When interpreting our study results, it is important to consider its limitations, quite common for online surveys, particularly those using snowball sampling, such as subjective reporting, potential recall and social desirability biases, and the inability to verify whether all the respondents were indeed Ukrainian war refugees due to the anonymity of the survey. Additionally, the online format may have constrained the complexity of questions, requiring simplifications that could impact the depth of the data collected. Finally, as a cross-sectional survey conducted on a convenience sample of European Ukrainian refugees, it may not have reached all population groups, limiting the generalizability of the findings to any single country or the entire EU. However, the study has notable strengths, providing robust evidence on refugees' access to essential healthcare services across the EU, particularly in terms of non-acute needs. This evidence can guide policy and decision-makers in better supporting refugees during and after the war in Ukraine. Furthermore, the methodology of the study and the questionnaire offer a solid foundation and a replicable tool for addressing health-related needs of future refugee populations entering Europe, while also strengthening the resilience of healthcare systems (38).

## Conclusions

Our survey highlights perceived gaps in healthcare services affecting Ukrainian war refugees, offering valuable insights for policymakers and organizations to design targeted interventions. These efforts are essential for mitigating the war's impact and reducing morbidity and mortality among this vulnerable group.

First and foremost, there is a need for better information provision in an easy-to-understand form, preferably in the Ukrainian language, covering key issues such as the scope of national health insurance, service costs and reimbursements, service availability, and registration procedures. Addressing these informational gaps could help minimize long waiting times, improve access, and lower costs.

Notably, managing chronic conditions is a growing concern, as many Ukrainian refugees will likely remain in EU countries for an extended period, increasing the demand for ongoing care. Chronic conditions have become increasingly significant, necessitating effective, long-term solutions. Experts emphasize the importance of continuous monitoring, organized health screenings, psychological trauma assessments, and culturally tailored care—all of which can be effectively delivered through robust primary healthcare services (15, 39–41). To ensure sustainable healthcare systems, system-wide decisions are crucial, enabling host countries to address the evolving needs of refugees while strengthening healthcare infrastructure for the future.

To sum up, the results of our study suggest several solutions for improving healthcare access for Ukrainian refugees. An integrated approach—combining better information, culturally competent care, and strengthened primary healthcare—provides a roadmap for enhancing national responses to the health needs of Ukrainian war refugees, ultimately benefiting both refugees and host populations.

## Data Availability

The raw data supporting the conclusions of this article will be made available by the authors, without undue reservation.

## Footnotes

^1^ https://eu-solidarity-ukraine.ec.europa.eu/information-people-fleeing-war-ukraine/fleeing-ukraine-healthcare_en
