# Supplementary material for: Barriers to healthcare access and continuity of care among Ukrainian war refugees in Europe: findings from the RefuHealthAccess study
Source: Front Public Health. 2025 Apr 2;13:1516161. doi: 10.3389/fpubh.2025.1516161 (PMC11999958; doi:10.3389/fpubh.2025.1516161)
Supplement: Supplementary file 1 [file Data_Sheet_1.pdf]

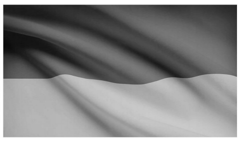

RefuHealthAccess Europe (technical name: UKR-2)

1. Introduction & informed consent

Welcome to the RefuHealthAccess Europe survey! Before providing your answers, please read carefully below information.

Participant Information

Study title: **Ukrainian War Refugee Access to Non-acute Healthcare Services Across Europe (RefuHealthAccess Europe)**

What is the project about and why do we ask you to participate?

RefuHealthAccess Europe is a scientific study focused on Ukrainian citizens who have been compelled to seek refuge in Europe due to the war initiated by Russia against Ukraine on February 24th, 2022. The primary objective of this study is to gain a deeper understanding of the healthcare experiences of Ukrainian refugees within Europe. Your valuable input will assist in examining the accessibility of the European healthcare system for Ukrainian war refugees, with the ultimate goal of formulating evidence-based policies to aid refugees like yourself in effectively addressing their health needs.

This study is a collaborative project of the ENABLE scientific partnership (www.enableadherence.eu), led by Medical University of Lodz. It has passed ethical procedures at Medical University of Lodz.

Description of the project

We prepared a questionnaire that we ask you to reply to.

Possible consequences and risks related to participation in the project

We did not identify any risks regarding your personal nor sensitive data, , nor contact information. You will not get any personal benefit, but your answers are expected to be helpful to identify potential problems of accessibility to the healthcare system and help to solve them.

What happens to my data?

Participation in this study is anonymous, and therefore we do not ask for your personal data. Sensitive data as age and gender will help to obtain a more complete picture in relation to the healthcare system accessibility.

How do I get information about the results of the project?

We plan to make results of this study public and generally available at the ENABLE web site (www.enableadherence.eu).

Voluntary participation

Your participation is purely voluntary. You may recall your consent at any moment.

Thank you for filling out this survey!

The RefuHealthAccess Europe Team:

Project leader & principal investigator:
Prof. Przemyslaw Kardas MD, PhD, Medical University of Lodz, Poland
Researchers:

- Tamas Agh MD, PhD University of Pecs, Hungary & Syreon Research Institute; Hungary
- Nilay Aksoy PharmD, PhD Altinbas University,Turkey
- Nataliia Khanyk PharmD, PhD Uppsala University, Sweden & Danylo Halytsky Lviv, National Medical University, Ukraine
- Marta Lomnytska, MD, Associate Professor, Institute of Women’s and Children’s Health, Academic Uppsala University Hospital, Uppsala University, Uppsala, Sweden
- Prof. Iryna Mogilevkina MD, PhD Uppsala University, Sweden & Bogomolets National Medical University, Ukrain
- Oksana Vorobiy, MD, Department of Obstetrics and Gynaecology, Academic Uppsala University Hospital, Uppsala, Sweden
- Prof. Björn Wettermark, MscPharm, PhD, Uppsala university, Sweden

* 1. Are you aged 18 years or more?

- Yes
- No

* 2. Being aware of the aims, terms and target results of the study, as well as fully anonymous nature of this survey, I voluntary:

- **Agree** to take part in this survey
- **Disagree** to take part in this survey

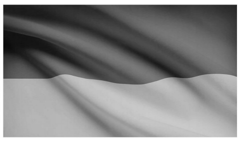

RefuHealthAccess Europe (technical name: UKR-2)

2. Your data

3. What is your **age** (in years)?

4. What is your **gender**?

- ◯ Male
- ◯ Female
- ◯ Other/not willing to provide

5. Which **country** do you **currently** live in?

Country

6. **How long** have you been living **in this country**? Please provide number of months.

* 7. **Are you a war refugee from Ukraine?**

- ◯ Yes
- ◯ No

RefuHealthAccess Europe (technical name: UKR-2)

3. Your data (2)

8. **Where** do you live?

- ◯ In a house/apartment
- ◯ Asylum centre
- ◯ Refugee Camp
- ◯ On the street
- ◯ Somewhere else (please specify)

9. What is your **residence status**?

- ◯ I am a citizen in this country
- ◯ I have Permanent documents (residence permit)
- ◯ I have Temporary documents (temporary protection)
- ◯ No documents/I am without legal documents
- ◯ Other (please specify)

10. What is your highest **educational level?**

- ◯ Primary school
- ◯ Secondary education
- ◯ Higher education
- ◯ No schooling
- ◯ Other

11. **With how many people** do you live under the same roof (please provide the number)?

12. **How many children** (0-18 years) are living with you?

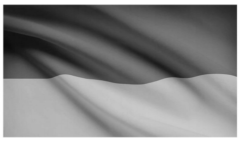

RefuHealthAccess Europe (technical name: UKR-2)

4. Availability of Information

13. How do you assess **ease of getting necessary information** (e.g. from web sites, phone info lines etc.) regarding the structure and operational procedures of the local healthcare system in this country?

- ◯ Very hard
- ◯ Hard
- ◯ Neither hard nor easy
- ◯ Easy
- ◯ Very easy
- ◯ Not applicable

14. Was the information on the structure and operational procedures of the local healthcare system in this country available **in Ukrainian language?**

- ◯ Not available at all
- ◯ Hardly available
- ◯ Neither hardly nor easily available
- ◯ Easily available
- ◯ Very easy available
- ◯ Not applicable

NOTE: below, we ask you for your experience within last twelve months (i.e. one year). However, please provide us with your experience limited to the **country of your current stay**, even if you stay there for a shorter period of time.

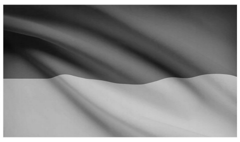

5. Acute illness

15. In the past twelve months in the country of your current stay, did you or another person in your household need to access health services for any **acute illness**?

- ◯ Yes
- ◯ No

6. Acute illness (2)

16. What is your, or this other person in your household experience regarding access to medical services for **acute illnesses**?

- ◯ Very poor access
- ◯ Poor access
- ◯ Neither poor nor good access
- ◯ Good access
- ◯ Very good access
- ◯ Not applicable

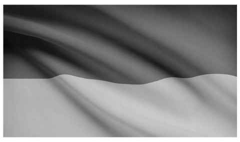

RefuHealthAccess Europe (technical name: UKR-2)

7. Chronic illness

17. In the past twelve months in the country of your current stay, did you or another person in your household need to access health services for any **chronic illness**?

- ◯ Yes
- ◯ No

RefuHealthAccess Europe (technical name: UKR-2)

8. Chronic illness (2)

18. What is your, or this other person in your household experience regarding access to medical services for **chronic illnesses**?

- ○ Very poor access
- ○ Poor access
- ○ Neither poor nor good access
- ○ Good access
- ○ Very good access
- ○ Not applicable

9. Child illness

19. In the past twelve months in the country of your current stay, did you or another person in your household need to access health services for any **child illness**?

- ◯ Yes
- ◯ No

RefuHealthAccess Europe (technical name: UKR-2)

10. Child illness (2)

20. What is your, or this other person in your household experience regarding access to medical services for **child illnesses**?

- ◯ Very poor access
- ◯ Poor access
- ◯ Neither poor nor good access
- ◯ Good access
- ◯ Very good access
- ◯ Not applicable

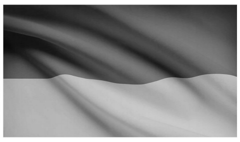

11. Sexual and reproductive care

21. In the past twelve months in the country of your current stay, did you or another person in your household need to access health **services for sexual and reproductive care (e.g. pregnancy, abortion or maternity care)?**

- ◯ Yes
- ◯ No

RefuHealthAccess Europe (technical name: UKR-2)

12. Sexual and reproductive care (2)

22. What is your, or this other person in your household experience regarding access to medical services for **sexual and reproductive care (e.g. pregnancy, abortion or maternity care)?**

- Very poor access
- Poor access
- Neither poor nor good access
- Good access
- Very good access
- Not applicable

RefuHealthAccess Europe (technical name: UKR-2)

13. Elderly care

23. In the past twelve months in the country of your current stay, did you or another person in your household need to access health services for **elderly care**?

- ◯ Yes
- ◯ No

14. Elderly care (2)

24. What is your, or this other person in your household experience regarding access to medical services for **elderly care**?

- ◯ Very poor access
- ◯ Poor access
- ◯ Neither poor nor good access
- ◯ Good access
- ◯ Very good access
- ◯ Not applicable

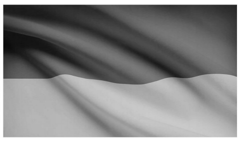

RefuHealthAccess Europe (technical name: UKR-2)

15. Psychological support and mental health

25. In the past twelve months in the country of your current stay, did you or another person in your household need to access health services for **psychological support and/or mental health?**

- ◯ Yes
- ◯ No

16. Psychological support and mental health (2)

26. What is your, or this other person in your household experience regarding access to medical services for **psychological support and/or mental health?**

- Very poor access
- Poor access
- Neither poor nor good access
- Good access
- Very good access
- Not applicable

17. Dental care

27. In the past twelve months in the country of your current stay, did you or another person in your household need to access health services for **dental care**?

- Yes
- No

RefuHealthAccess Europe (technical name: UKR-2)

18. Dental care (2)

28. What is your, or this other person in your household experience regarding access to medical services for **dental care?**

- ◯ Very poor access
- ◯ Poor access
- ◯ Neither poor nor good access
- ◯ Good access
- ◯ Very good access
- ◯ Not applicable

19. Vaccination

29. In the past twelve months in the country of your current stay, did you or another person in your household need to access health services for **vaccination**?

- ◯ Yes
- ◯ No

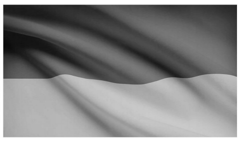

20. Vaccination (2)

30. What is your, or this other person in your household experience regarding access to medical services for **vaccination**?

- ◯ Very poor access
- ◯ Poor access
- ◯ Neither poor nor good access
- ◯ Good access
- ◯ Very good access
- ◯ Not applicable

RefuHealthAccess Europe (technical name: UKR-2)

21. Cancer care

31. In the past twelve months in the country of your current stay, did you or another person in your household need to access health services for **cancer care?**

- ◯ Yes
- ◯ No

22. Cancer care (2)

32. What is your, or this other person in your household experience regarding access to medical services for **cancer care**?

- ◯ Very poor access
- ◯ Poor access
- ◯ Neither poor nor good access
- ◯ Good access
- ◯ Very good access
- ◯ Not applicable

RefuHealthAccess Europe (technical name: UKR-2)

23. Cancer screening

33. In the past twelve months in the country of your current stay, did you or another person in your household need to use any sort of **cancer screening (e.g. cervical cancer, breast cancer, testicular cancer, colon cancer)?**

- ◯ Yes
- ◯ No

24. Cancer screening (2)

34. What is your, or this other person in your household experience regarding access to **cancer screening (e.g. cervical cancer, breast cancer, testicular cancer, colon cancer)?**

- ◯ Very poor access
- ◯ Poor access
- ◯ Neither poor nor good access
- ◯ Good access
- ◯ Very good access
- ◯ Not applicable

RefuHealthAccess Europe (technical name: UKR-2)

25. Prescription drugs

35. In the past twelve months in the country of your current stay, did you or another person in your household need to use any sort of **prescription drugs?**

- ◯ Yes
- ◯ No

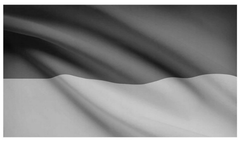

26. Prescription drugs (2)

36. What is your, or this other person in your household experience regarding access to **prescription drugs?**

- ◯ Very poor access
- ◯ Poor access
- ◯ Neither poor nor good access
- ◯ Good access
- ◯ Very good access
- ◯ Not applicable

27. Obstacles toward health services

37. How important in your case, or case of persons living in your household, were these **obstacles toward health services** within last 12 months?

	Very important	Important	Neither important nor not important	Not important	Not important at all	Not Applicable
Information barrier (e.g. lack of information, language, cultural barrier)	○	○	○	○	○	○
High cost of the service	○	○	○	○	○	○
Logistic difficulties (transport, distance)	○	○	○	○	○	○
Registration procedures	○	○	○	○	○	○
Long waiting time	○	○	○	○	○	○
Lack of coverage with national insurance of the host country	○	○	○	○	○	○

38. If you experienced **any additional obstacles** while trying to access the health services you needed in the past twelve months in the country of your current stay, please provide the details in the box.

39. If in the past twelve months you needed to go back to Ukraine temporarily, just in order to get access to any healthcare service you needed, and **could not access in your country of current residence,** please provide the details of the reason in the box.

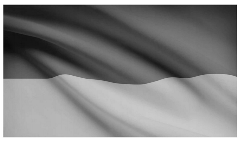

RefuHealthAccess Europe (technical name: UKR-2)

28. Thank you!

Thank you for taking part in this survey!

Please share the link to this survey with at least 10 other Ukrainian people being currently refugees to one of the European countries!

Here is the link you may copy: XXXXXXXXXXXX

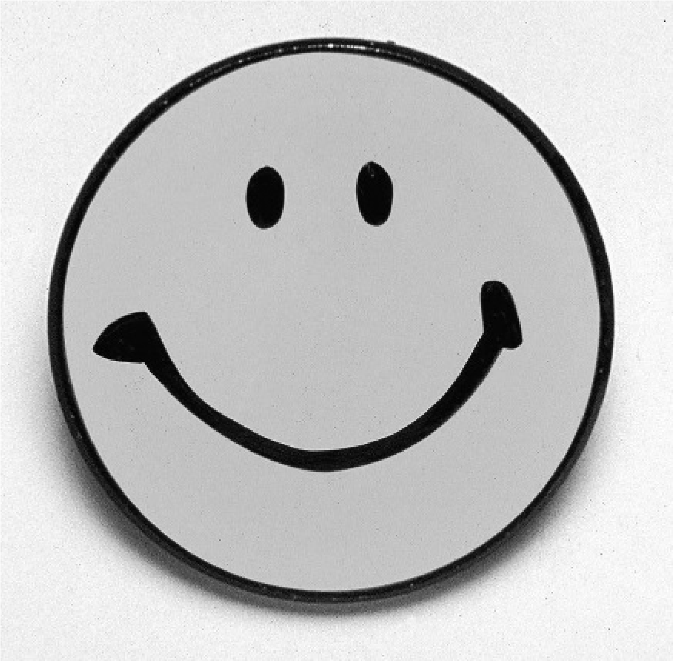

More information on the access to the healthcare services in European countries you can find here: https://eu-solidarity-ukraine.ec.europa.eu/information-people-fleeing-war-ukraine/fleeing-ukraine-healthcare_enlink
