# Supplementary material for: Barriers to healthcare access and continuity of care among Ukrainian war refugees in Europe: findings from the RefuHealthAccess study
Source: Front Public Health. 2025 Apr 2;13:1516161. doi: 10.3389/fpubh.2025.1516161 (PMC11999958; doi:10.3389/fpubh.2025.1516161)
Supplement: Supplementary file 2 [file Data_Sheet_2.pdf]

**Online Material 2**

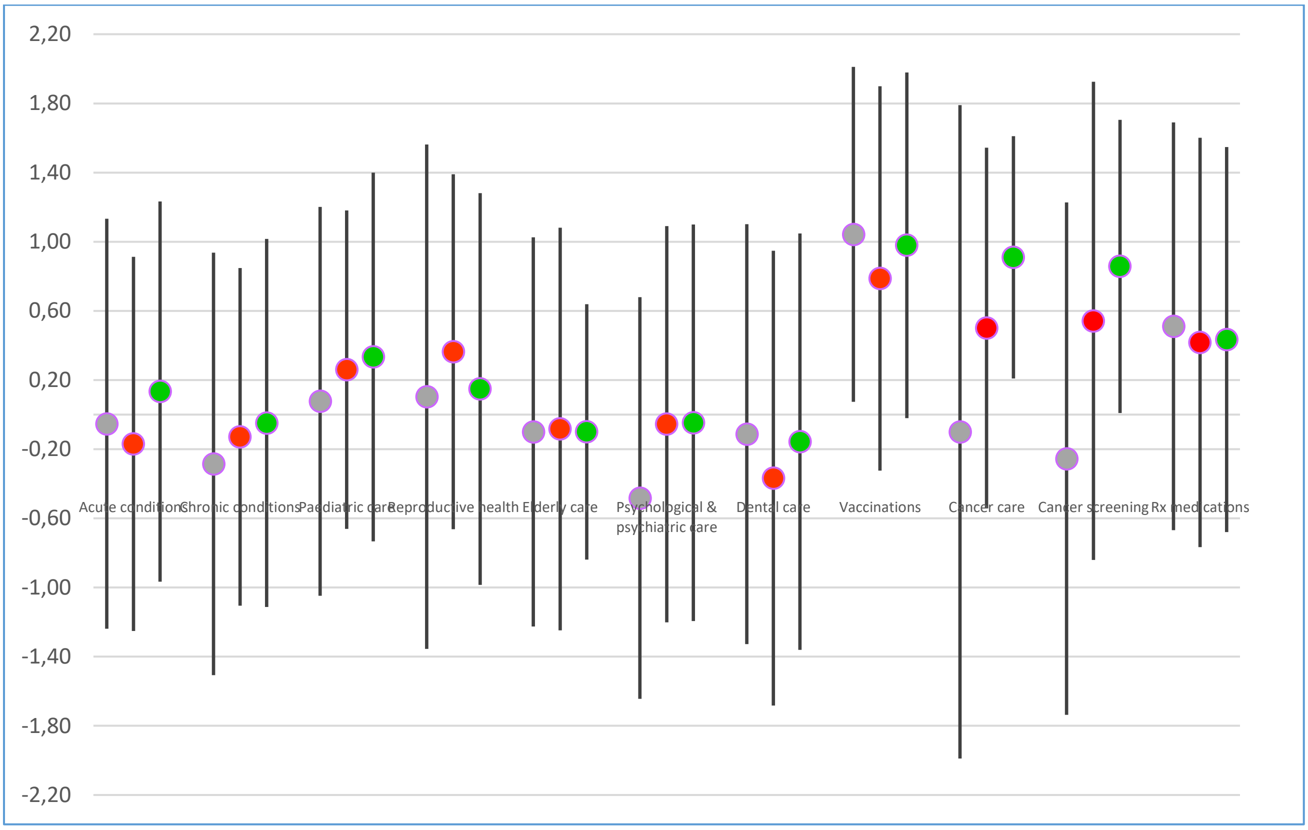


Access to various health services (either for the study participants or their household members) across the benchmarked countries, as reported by the respondents. The figure presents mean values of access score +/- standard deviation (SD), calculated for values ascribed to Likert scale answers (where -2 corresponds to 'very poor access', 1 to 'poor access', 0 to 'neither poor nor good access', 1 to 'good access' and 2 to 'very good access')

Sweden – purple dots, Poland – red dots, Lithuania – green dots.
