# Supplementary material for: Barriers to healthcare access and continuity of care among Ukrainian war refugees in Europe: findings from the RefuHealthAccess study
Source: Front Public Health. 2025 Apr 2;13:1516161. doi: 10.3389/fpubh.2025.1516161 (PMC11999958; doi:10.3389/fpubh.2025.1516161)
Supplement: Supplementary file 3 [file Data_Sheet_3.PDF]

Online Material 3

	Lithuania		Poland		Sweden	
	**N**	**%***	**N**	**%***	**N**	**%***
Dental care	19	12.5	11	12.1	48	19.5
Gynecology	4	2.6	7	7.7	21	8.5
Ophthalmology	0	0.0	0	0.0	0	0.0
Endocrinology	1	0.7	1	1.1	7	2.8
Orthopedics	0	0.0	0	0.0	0	0.0
Otolaryngology	0	0.0	0	0.0	0	0.0
Dermatology	0	0.0	1	1.1	2	0.8
Gastroenterology	0	0.0	0	0.0	0	0.0
Neurology	1	0.7	1	1.1	2	0.8
Other#	6	3.9	5	5.5	24	9.8
Respondents needing to go back to Ukraine	27	17.8	22	24.2	69	28.0
**Total number of respondents**	**152**	**100.0**	**91**	**100.0**	**246**	**100.0**

Healthcare services that the respondents indicated as those unobtainable in their current country of residence, which prompted them to temporarily return to Ukraine for access within the previous twelve months.

* The percentage rates are calculated based on the total number of respondents per country. Note that many respondents indicated multiple services as unavailable.

# It also included various diagnostic procedures.
